# Components and Public Health Impact of Population Growth in the Arab World

**DOI:** 10.1371/journal.pone.0124944

**Published:** 2015-05-18

**Authors:** Asharaf Abdul Salam, Ibrahim Elsegaey, Rshood Khraif, Abdullah AlMutairi, Ali Aldosari

**Affiliations:** Center for Population Studies, King Saud University, Riyadh, Saudi Arabia; National Institute of Health, ITALY

## Abstract

The Arab world, which consists of the 22 member states of the Arab League, is undergoing a rapid transition in demographics, including fertility, mortality, and migration. Comprising a distinctive geographic region spread across West Asia and North East Africa and unified by the Arabic language, these states share common values and characteristics despite having diverse economic and political conditions. The demographic lag (high fertility and low mortality) that characterizes the Arab world is unique, but the present trend of declining fertility, combined with the relatively low mortality, brings about significant changes in its population size. This research aimed to: (i) assess the population growth in the Arab world over 3 time periods, (ii) explore its components, and (iii) understand its public health impact. Data from the International Data Base (IDB) of the U.S. Census Bureau for 3 time periods (1992, 2002, and 2012) in 21 countries of the Arab world were analyzed by dividing them into four geographic sectors, namely, the Gulf Cooperation Council (GCC), West Asia, Maghreb, and the Nile Valley African Horn. The population of the Arab world has grown considerably due to both natural growth and migration. The immigration is pronounced, especially into resource-intensive GCC nations, not only from East Asian and Central African countries but also from resource-thrifty (limited-resource) Arab nations. The migrations within, as well as outside, the Arab world reveal an interesting demographic phenomenon that requires further research: migration flows and trends. However, the transformations in public health statistics related to mortality—the impact of demographic changes—depict a new era in the Arab world.

## Introduction

The Arab world has been undergoing transitions [1,2,3] in all fields of life—social, economic, and health—due to changing demographic conditions [4,5]. The achievement of low mortality [1,6] as a result of improvements in medical technologies [4], housing, water quality, sanitation, electric supply, public hygiene, health, and educational infrastructure [7] leads to improved health and quality of life [8], which in turn facilitates values (social and economic), as well as a desire to bring children up with great hope [4]—a sign of fertility transition [2,9,10]. The resultant improvement in population health leads to socioeconomic benefits, as seen in less developed countries [4]. The demographic transition theory propounds that a large increase in population is due to the gap between birth and death rates during the early stages of industrialization, urbanization, and socioeconomic transformation; this seems to play a role in what is happening in the Arab world today [1,7,9].

A distinctive region both geographically and demographically, the Arab world is spread across two continents (Asia and Africa), has a common language and lifestyle and is coordinated by the League of Arab Nations. Having two thirds of the known petroleum reserves worldwide and given the fast pace of its modernization, urbanization, and economic transformation, the region experiences a rapid growth in population [1], due partly to a natural increase and partly to migration; the latter refers to both internal migration, including inter-Arab movements [5] within the region, and employment-oriented migration from elsewhere [6,11].

The resultant demographic dividend and youth bulge in the Arab world [1,12], characterized by a demographic lag—high fertility and low mortality [13,14]—put pressure on social, economic, and political institutions to capitalize on the growing pool of potential workers by expanding educational systems, labor markets, housing supply, and health systems to adapt to the needs of people and national economies [1,3]. This, in turn, exerts pressure to decrease the fertility rate faster [2], which is influenced by another set of variables: school enrolment of girls, participation of women in the labor force, wait-hood (delay) in marriage, and formation of smaller families [9]. Thus, the rapid population growth in the Arab world since the 1950s puts pressure on the labor market, education, housing, health, and other public services that influence family formation and future population growth, toward reducing the youth population in the future [15,16,17] while promoting life expectancy [18,19].

The demographic lag exaggerates population growth because the fast transition in mortality rates is not accompanied by a transition in fertility [1,3,6], despite having economic and social development consequences that influence life span and human welfare [4,20]. Thus, population growth is affected by the speed of transition in fertility and mortality, in addition to the associated economic and political changes that determine population movement and urbanization [9]. Components of population growth, namely, natural increase and net migration, have a bearing not only on the demographic transition but also on the socioeconomic and infrastructural development in a country. The Arab world, a union of 22 member states spread across a vast geographic area, experiences natural growth and net migration in varying degrees [6,9] depending on the socioeconomic infrastructure.

The slow pace of demographic transition creates footprints on vital statistics [2], namely, demographic and public health indicators, in turn offering improvements in quality of life, administrative infrastructure, and efficiency of utility networking [4], as an improvement in the population profile results from a combination of variables. Arab countries as a whole progressed remarkably during the second half of the previous century [1,5], as reflected in the reproduction, infant and child mortality, and life expectancy rates. The rapid fall in birth rates [11] as a result of changes in lifestyle—age at marriage, female education and employment, urbanization, nucleation of families, and value systems [1]—signals a new era of demographic revolution (a series of research and development efforts leading to fertility decisions, mortality control, migration laws and regulations, and healthy life expectancy) in the Arab world.

The research questions addressed by this paper are: (i) Is the Arab world witnessing a rapid population growth in tune with the high fertility/low mortality scenario?, (ii) How do the two components (natural increase and migration) operate together to increase the population of the Arab world?, (iii) What interrelationships exist between population growth and public health?, and (iv) Does the increase in population put pressure on other sectors of public health and lifestyle, leading to a realization of, and thus concerted efforts toward, improved living conditions that facilitate healthy life expectancy?

## Objectives

This research aims to: (i) analyze changes in population size in the Arab world since 1992, (ii) assess the components of the population growth, and (iii) explore changes in public health statistics over the specified study period.

## Methodology

This analysis is based on the International Data Base (IDB) of the U.S. Census Bureau [21] for 3 time periods: 1992, 2002, and 2012 (accessed in June to July 2012). Of the 22 Arab countries included in the Arab League, only Palestine has no data recorded on the IDB. The Arab countries are spread over two continents, Asia and Africa. Those in Asia are divided into Gulf Cooperation Council (GCC) members and nonmembers (West Asia). The six member countries of the GCC include Bahrain, Kuwait, Oman, Qatar, Saudi Arabia, and the United Arab Emirates. The West Asian countries include Iraq, Jordan, Lebanon, Syria, and Yemen. Those in the African continent are divided into Maghreb and others (the Nile Valley African Horn). The Maghreb countries include Algeria, Libya, Mauritania, Morocco, and Tunisia. The Nile Valley African Horn includes Comoros, Djibouti, Egypt, Somalia, and Sudan. This classification has relevance because it reflects geographic, economic, developmental, and infrastructural dimensions as against the classification into three groups on the basis of fertility level [17].

Data on Oman for the year 1992 were not available; thus, 1993 data were used. Similarly, data of on Yemen for 1992 and 1993 were not available, so 1994 data were used instead. Data on Egypt from 1992 to 1995, were also not available, so 1996 data were applied in the analysis. Data on Sudan up to the year 2000 were not available, except for total population. The following calculations [22,23,24] were made with the raw data:

- Population growth rate, the exponential growth rate calculated by using the formula:

r=(ln(Pn÷P0))÷n

Where: P_n_ = population at the last census

P_0_ = population at the previous census

ln = natural logarithms

n = intercensal period

- Natural increase, the difference between births and deaths
- Population change, the sum of net migrants (inmigrants-outmigrants) and natural increase

Efforts were made to interpret fertility rates, including the crude birth rate (the number of births in a year per 1000 midyear population—CBR), total fertility rate (the sum of all births to a woman during her childbearing age, often defined as ages 15–49 years—TFR), and gross reproduction rate (the number of daughters a woman gives birth to during her childbearing period—GRR), as well as mortality rates, including the crude death rate (the number of deaths in a year per 1000 midyear population—CDR), infant mortality rate (the number of deaths of infants (less than one year old) in a year per 1000 live births—IMR), under-5 mortality rate (the number of deaths of children below 5 years old in a year per 1000 children below 5 years old—U5MR), and expectation of life at birth (the expected number of years to be lived, on average, by a newborn at a particular time—e^0^_0_) [25,26,27,28]: these vital statistics reflect the public health scenario in a given population. All the rates were calculated by the U.S. Census Bureau and provided on their online database.

In addition, the CBR, TFR, and CDR for the Arab world (21 countries together) and for its four sectors were calculated with the PAS software by using the birth rates for individual states which were available at the source. The TFR for the sectors and the region were calculated based on the number of births and the age-sex distribution of the population.

## Results and Discussion

The population of the Arab world grew from 232 million in 1992 to 360 million in 2012. Of the four sectors, the Nile Valley African Horn has the biggest population, possibly due to its having the largest land area and the large population of Egypt and Sudan. The Nile Valley African Horn accounts for 39.3 percent of the total population of the Arab world. The GCC represents 11.3 percent; West Asia, 24.7 percent; and Maghreb, 24.6 percent.

### Population Growth

The Arab world is recognized as a region with a growing population due to the demographic lag—low death rate and high birth rate [6,13,14]. From 1992 to 2012, the population experienced a huge increase (Table 1), which occurred in conjunction with a continuing decline in fertility [1,4,5]. The higher increase in 2002–2012 (76.0 million) compared with 1992–2002 (51.5 million) indicates a quantum of change in the region (an overall increase of 127.5 million people). The window of opportunity brought about by the age structural transition reduces the dependency ratio and increases the working-age population, a demographic bonus resulting from the large supply of human capital [1,12,20] and promoted by the goal of near-replacement fertility levels by half of the Arab countries by 2025 [6]. Gender-wise, the male population increased more than the female population throughout the period; this is explained by the intense male-dominated labor migration from East Asia and Central Africa, especially into GCC states [11], which may alter in the near future with the changing labor laws. A wide growth gap of 3.1 million (1992–2012) between male and female populations was observed in GCC nations. Similar gaps do not exist in the other sectors, indicating that GCC states have a wider gender gap.

**Table 1:** Population growth in the Arab world between 1992 and 2012.

Sectors and States	1992–2002			2002–2012			1992–2012			Difference between 2002–2012 and 1992–2002		
	Male	Female	Total	Male	Female	Total	Male	Female	Total	Male	Female	Total
GCC												
Bahrain	102323	74488	176811	345755	187762	533517	448078	262250	710328	243432	113274	356706
Kuwait	442649	237935	680584	324272	238737	563009	766921	476672	1243593	-118377	802	-117575
Oman	255313	271199	526512	262290	286782	549072	517603	557981	1075584	6977	15583	22560
Qatar	159900	79976	239876	1031074	217130	1248204	1190974	297106	1488080	871174	137154	1008328
Saudi Arabia	2925953	2287336	5213289	2046648	2213817	4260465	4972601	4501153	9473754	-879305	-73519	-952824
UAE	1061328	440481	1501809	1221392	534827	1756219	2282720	975308	3258028	160064	94346	254410
Total	4947466	3391415	8338881	5231431	3679055	8910486	10178897	7070470	17249367	283965	287640	571605
West Asia												
Iraq	3126740	3009032	6135772	3620536	3504642	7125178	6747276	6513674	13260950	493796	495610	989406
Jordan	498480	532665	1031145	784992	821840	1606832	1283472	1354505	2637977	286512	289175	575687
Lebanon	127397	160034	287431	143604	161222	304826	271001	321256	592257	16207	1188	17395
Syria	2005200	1982971	3988171	2638674	2592254	5230928	4643874	4575225	9219099	633474	609283	1242757
Yemen	2079988	2071465	4151453	3123071	3102535	6225606	5203059	5174000	10377059	1043083	1031070	2074153
Total	7837805	7756167	15593972	10310877	10182493	20493370	18148682	17938660	36087342	2473072	2426326	4899398
Maghreb												
Algeria	2562779	2467303	5030082	1891083	1991509	3882592	4453862	4458812	8912674	-671696	-475794	-1147490
Libya	459741	474873	934614	729942	758554	1488496	1189683	1233427	2423110	270201	283681	553882
Mauritania	280877	308152	589029	349462	377878	727340	630339	686030	1316369	68585	69726	138311
Morocco	1883482	2094335	3977817	1633783	1836561	3470344	3517265	3930896	7448161	-249699	-257774	-507473
Tunisia	577264	618481	1195745	477417	545262	1022679	1054681	1163743	2218424	-99847	-73219	-173066
Total	5764143	5963144	11727287	5081687	5509764	10591451	10846830	11472908	22318738	-682456	-453380	-1135836
Nile Valley African Horn												
Comoros	58784	64959	123743	77759	85055	162814	136543	150014	286557	18975	20096	39071
Djibouti	68644	100622	169266	21600	36438	58038	90244	137060	227304	-47044	-64184	-111228
Egypt	4054806	4210495	8265301	7734420	7930620	15665040	11789226	12141115	23930341	3679614	3720125	7399739
Somalia	910438	990106	1900544	1054603	1014278	2068881	1965041	2004384	3969425	144165	24172	168337
Sudan	-	-	5379953	-	-	18023033	-	-	23402986	-	-	12643080
Total	5092672	5366182	15838807	32045040	32093095	35977806	37137712	37459277	51816613	26952368	26726913	20138999
Arab World	23642086	22476908	51498947	52669035	51464407	75973113	76311121	73941315	127472060	29026949	28987499	24474166

**Fig 1:**
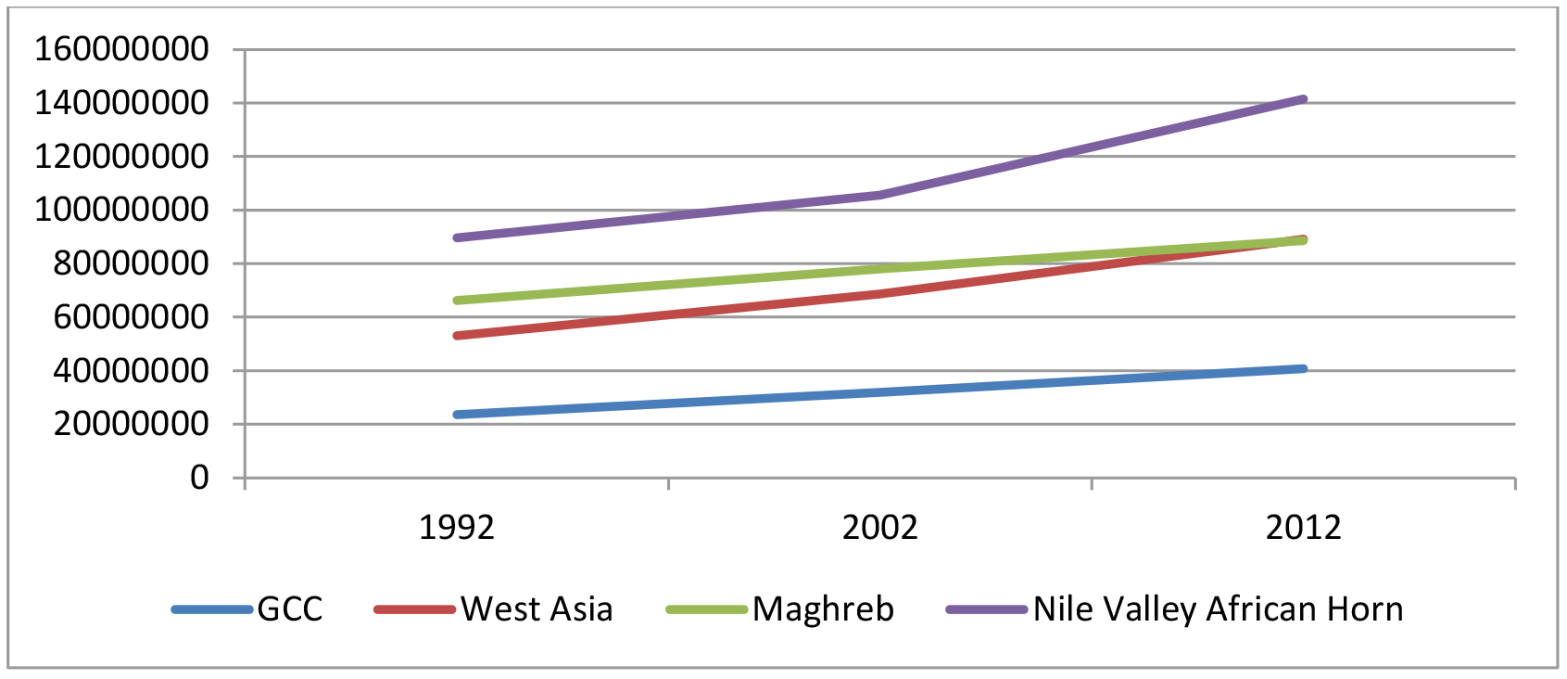
Population growth in the Arab world by sector.

The Nile Valley African Horn reported the highest population growth (52 million), followed by West Asia (36 million), Maghreb (22 million), and the GCC (17 million). Egypt and Sudan accounted for most of the population growth in the Nile Valley African Horn (24 million and 23 million, respectively), as did Iraq, Yemen, and Syria in West Asia (13 million, 10 million, and 9 million, respectively), Saudi Arabia in the GCC (9 million), and Algeria and Morocco in Maghreb (9 million and 7 million, respectively). Thus, each sector has at least one member country with a large population. Overall, the growth gap between male and female populations is 2.4 million, with males increased by 76.3 million and females by 73.9 million (24 million and 22 million, respectively, for males and females in 1992–2002; 53 million and 51 million, respectively, for males and females in 2002–2012). The population growth favored males in the GCC and West Asia, and females in Maghreb and the Nile Valley African Horn (Fig 1).

The population of the region grew at an annual rate of 2.19 percent during 1992–2012, which means that 2 persons were added for every 100 persons annually (Table 2), despite a downward trend from 3.0 (1980–85) to 2.1 percent (1995–2000), intermittently affected by migration [6]. The female population grew at a faster rate (2.73) than the male population (2.67). Growth rates increased comparatively in 2002–2012 and remained higher in the Asian sectors; both the male and female populations were higher in the GCC (2.75) than in West Asia (2.60). The male population registered a higher growth rate in the GCC, whereas the female population grew faster in West Asia. Annual growth rates were slightly higher in the GCC during 1992–2002 but reduced further during 2002–2012. Higher growth rates prevailed in Qatar, the UAE, and Bahrain.

**Table 2:** Rate of population growth in the Arab world between 1992 and 2012.

Sectors andstates	1992–2002			2002–2012			1992–2012			Difference between 2002–2012 and 1992–2002		
	Male	Female	Total	Male	Female	Total	Male	Female	Total	Male	Female	Total
GCC												
Bahrain	2.86	2.81	2.84	6.10	4.81	5.58	4.48	3.81	4.21	3.24	2.00	2.74
Kuwait	4.46	3.27	3.96	2.34	2.47	2.39	3.40	2.87	3.17	-2.12	-0.80	-1.57
Oman	1.96	2.81	2.32	1.68	2.30	1.96	1.82	2.55	2.14	-0.28	-0.51	-0.36
Qatar	4.21	4.10	4.17	11.68	6.49	10.2	7.94	5.29	7.19	7.47	2.39	6.03
Saudi Arabia	2.67	2.66	2.67	1.52	2.04	1.75	2.10	2.35	2.21	-1.15	-0.62	-0.92
UAE	5.74	4.94	5.48	4.07	3.88	4.01	4.91	4.41	4.75	-1.67	-1.06	-1.47
Total	3.12	2.91	3.03	2.50	2.42	2.47	2.81	2.66	2.75	-0.62	-0.49	-0.56
West Asia												
Iraq	2.98	2.93	2.95	2.61	2.59	2.60	2.79	2.76	2.78	-0.37	-0.34	-0.35
Jordan	2.20	2.53	2.36	2.71	2.97	2.84	2.46	2.75	2.6	0.51	0.44	0.48
Lebanon	0.70	0.86	0.78	0.73	0.79	0.76	0.72	0.83	0.77	0.03	-0.07	-0.02
Syria	2.58	2.66	2.62	2.62	2.66	2.64	2.60	2.66	2.63	0.04	0	0.02
Yemen	2.49	2.58	2.53	2.86	2.93	2.89	2.67	2.76	2.71	0.37	0.35	0.36
Total	2.55	2.61	2.58	2.59	2.63	2.61	2.57	2.62	2.6	0.04	0.02	0.03
Maghreb												
Algeria	1.75	1.72	1.74	1.12	1.20	1.16	1.44	1.46	1.45	-0.63	-0.52	-0.58
Libya	1.86	2.08	1.96	2.38	2.62	2.50	2.12	2.35	2.23	0.52	0.54	0.54
Mauritania	2.50	2.56	2.53	2.43	2.45	2.44	2.47	2.50	2.49	-0.07	-0.11	-0.09
Morocco	1.42	1.55	1.48	1.08	1.19	1.14	1.25	1.37	1.31	-0.34	-0.36	-0.34
Tunisia	1.26	1.37	1.31	0.93	1.07	1.00	1.10	1.22	1.16	-0.33	-0.30	-0.31
Total	1.60	1.66	1.63	1.22	1.32	1.27	1.41	1.49	1.45	-0.38	-0.34	-0.36
Nile Valley African Horn												
Comoros	2.36	2.49	2.43	2.45	2.54	2.50	2.40	2.51	2.46	0.09	0.05	0.07
Djibouti	2.29	3.07	2.70	0.62	0.91	0.78	1.45	1.99	1.74	-1.67	-2.16	-1.92
Egypt	1.24	1.35	1.30	2.02	2.13	2.07	1.63	1.74	1.68	0.78	0.78	0.77
Somalia	2.58	2.84	2.71	2.34	2.26	2.30	2.46	2.55	2.50	-0.24	-0.58	-0.41
Sudan	-	-	2.12	-	-	4.95	-	-	3.53	-	-	2.83
Total	1.39	1.52	1.63	5.97	6.11	2.93	3.68	3.82	2.28	4.58	4.59	1.30
Arab World	1.98	2.0	2.0	3.37	3.47	2.37	2.67	2.73	2.19	1.39	1.47	0.37

Saudi Arabia and Oman, among other countries, reported low growth rates during 2002–2012. West Asia showed equal and moderate growth rates during both periods. Whereas Lebanon experienced lower growth rates, all other countries showed annual growth rates of nearly 3 per 100 persons for both sexes. Maghreb experienced a lower annual growth rate compared with the GCC and West Asia. Mauritania, which is among the Maghreb countries, registered the highest annual growth rate, whereas Tunisia had the lowest. The Nile Valley African Horn had a lower growth rate; this was particularly attributed to Egypt because Djibouti and Somalia had higher growth rates. A declining trend in growth rate was observed in the GCC and Maghreb; in contrast, an increasing trend was seen in West Asia and the Nile Valley African Horn. Thus, the overall increase in growth rate was determined largely by the population size.

### Components of Population Growth

Population change occurred in two ways, namely: (i) through natural increase, the difference between the number of births and the number of deaths in a given population; and (ii) through net migration, the difference between the number of immigrants and the number of emigrants at a given point in time. The overall contributions of natural increase and net migration add up to the existing population, thus contributing to growth.

An analysis of the population change during the last two decades (1993–2002 and 2003–2012) led to the delineation of major developments. According to the U.S. Census Bureau, one of the limitations in interpreting migration flow is the lack of data on place of birth and place of residence, which makes it difficult to understand inter-Arab migration. The region had a higher migration flow from one country to another such that some countries pull in (import labor) whereas others push out (export labor) people [1,6,9,29].

Table 3 shows that during 2003–2012, 2.2 million people out-migrated, whereas 63.9 million people were added due to natural increase. In 1993–2002, 58.9 million people were added—58.7 million through natural increase and 0.2 million through immigration. Although these data cannot explain the population growth for the region as a whole or for the sectors within the region, they explain the population growth in each country. For example, the population change in GCC countries during 1993–2002 and 2003–2012 was due more to immigration than to natural increase [11], especially in Qatar and the UAE. Bahrain and Kuwait had higher growth rates due to immigration during 2003–2012. Thus, the pull factor influenced population growth in the GCC during 2003–2012, except in Oman and Saudi Arabia. The push factor operated in all other countries except Iraq, Syria, and Djibouti during 1993–2002 and except Jordan during 2003–2012. Emigration from some countries, namely, Iraq, Libya, Egypt, Tunisia, and Syria, due to recent political crises also merited attention. The migration trend from rural to urban strengthens urbanization in the Arab world, especially in Kuwait, Qatar, Bahrain, and Lebanon [9,30]. The migrations are classified as adjustment, induced, or forced, and are due to such reasons as house purchase, family size issues, rental issues, and the search for better dwelling or services [31].

**Table 3:** Components of population growth in the Arab world.

Sectors and states	1993–2002					2003–2012				
	Births	Deaths	Net migrants	Natural Increase	Population change	Births	Deaths	Net migrants	Natural Increase	Population change
GCC										
Bahrain	142575	19932	79887	122640	202530	165520	27741	379427	137778	517204
Kuwait	414300	39825	292147	374477	666624	525086	51966	86763	473122	559882
Oman	650613	92610	26344	558003	584350	664471	100171	-10244	564299	554056
Qatar	109715	11871	150369	97843	248211	161929	22116	1140528	139813	1280341
Saudi Arabia	5633399	780518	307251	4852884	5160132	5143434	844930	-73028	4298506	4225477
UAE	510309	70553	1085660	439758	1525415	726693	96920	1121637	629774	1751411
Total	7460911	1015309	1941658	6445605	8387262	7387133	1143844	2645083	6243292	8888371
West Asia										
Iraq	7549899	1370574	27004	6179326	6206330	8602681	1450321	0	7152360	7152360
Jordan	1335958	113240	-193981	1222716	1028733	1627649	151058	43758	1476590	1520349
Lebanon	616383	254250	-75442	362134	286693	701536	221153	-201710	480383	278673
Syria	5296835	769055	405852	4527781	4933631	4936783	658790	-270129	4277992	4007866
Yemen	6245664	1544392	-15598	4701271	4685672	7964473	1710830	0	6253643	6253643
Total	24108021	4335514	374168	19772508	20146676	20769840	3908149	-654414	16861688	16207274
Maghreb										
Algeria	6595155	1470380	-224220	5124772	4900551	7911838	1500723	-429829	6411115	5981285
Libya	1222682	204466	-82128	1018212	936085	1163854	234660	-371642	929196	557553
Mauritania	921627	280303	-63574	641322	577746	1047351	284403	-29987	762949	732959
Morocco	6605320	1386551	-1276512	5218770	3942258	6203927	1461155	-1282492	4742773	3460281
Tunisia	1769152	497899	-102886	1271252	1168367	1778098	586128	-167738	1191970	1024231
Total	17113936	3839599	-1749320	13274328	11525007	18105068	4067069	-2281688	14038003	11756309
Nile Valley African Horn										
Comoros	202207	56059	-19753	146149	126395	244178	61666	-1970	182512	162749
Djibouti	226508	62944	41735	163567	205302	176608	55660	-81036	120948	39913
Egypt	12192241	2397129	-149154	9795111	9645957	19679021	3772014	-168534	15907006	15738474
Somalia	3184813	1437475	427530	1747337	2174868	4106083	1484311	-596084	2621772	2025687
Sudan	10807017	3389680	-688834	7417342	6728504	11328296	3454028	-1038906	7874268	6835362
Total	26612786	7343287	-388476	19269506	18881026	35534186	8827679	-1804321	26706506	24802185
Arab World	75295654	16533709	178030	58761947	58939971	81815517	17952998	-2190678	63862522	61671834

The Arab world experiences higher birth rates but lower death rates, resulting in a higher natural increase in population [1]. Compared with 58.9 million in 1993–2002, the natural increase during 2003–2012 was higher at 63.9 million; the Nile Valley African Horn had the highest rate (26,706,506), followed by West Asia **(**16,861,688), Maghreb (14,038,003), and the GCC (6,243,292). The natural increase was lesser during 1993–2002, with 58,761,947 persons added to the whole region: the highest increase was in West Asia (19,772,508), followed by the Nile Valley African Horn (19,269,506), Maghreb (13,274,328), and the GCC (6,445,605). Egypt (15,907,006), Sudan (7,874,268), Algeria (6,411,115), Yemen (6,253,643), Saudi Arabia (4,298,506), and Syria (4,277,992) registered high rates of natural increase. Bahrain (137,778), Comoros (182,512), and Lebanon (480,383) had low rates of natural increase, which the rest of the Arab world intends to follow as they recognize the importance of population growth restriction [3]. Egypt (9,795,111), Sudan (7,417,342), Iraq (6,179,326), Algeria (5,124,772), Saudi Arabia (4,852,884), Yemen (4,701,271), and Syria (4,527,781) also had high rates of natural increase during 1993–2002.

### Public Health Impact of Population Growth

Vital statistics relating to both fertility and mortality were considered in exploring the impact of population growth. Changes in fertility and mortality were interpreted as impacts of population growth that influence the public mental set, leading to a realization of population pressures and thereby molding attitudes toward healthy behaviors. Fertility indicators, namely, the crude birth rate, total fertility rate, and general reproduction rate, as well as mortality indicators, namely, the crude death rate, infant mortality rate, under-5 mortality rate, and expectation of life at birth, reflect such a realization.

### Fertility

The high level of fertility in Arab countries has been the subject of debate. There is generally a declining trend [1,3] depending on the pace of development and quality of life. For example, GCC countries improved in terms of socioeconomic conditions and developed lifestyles comparable with those in modernized states; thus, they experienced a higher decline in fertility rates [32] despite the uncertain availability and use of contraception and abortion services [4]. Fertility indicators, namely, the crude birth rate, total fertility rate, and general reproduction rate, registered a decline (Table 4). Whereas the CBR declined from around 25 (1992) to around 15 (2012), the TFR declined from around 4.0 (1992) to around 2.5 (2012), a reflection of the acceptance of population growth restriction by the Arab world [3,33] in line with the Millennium Development Goals [34,35]. The fertility indicators in West Asia were promising, with Lebanon having the lowest levels since 1992 [36], which in turn attracted nearby Syria to follow the trend. Yemen had a higher fertility rate even in 2012, followed by Iraq and Jordan. The Maghreb countries reported higher fertility levels during 1992, but these declined in 2002 and further in 2012; among them, Mauritania had the highest fertility rate, followed by Libya. Age at marriage and female education, which are determinants of fertility [37], improved in the region, along with a decline in son preference and child deaths.

**Table 4:** Indices related to fertility.

Sectors and States	1992*			2002			2012		
	CBR	TFR	GRR	CBR	TFR	GRR	CBR	TFR	GRR
GCC									
Bahrain	25.8	3.4	1.7	19.5	2.4	1.2	14.0	1.9	0.9
Kuwait	24.8	3.4	1.7	20.9	2.6	1.3	21.0	2.6	1.3
Oman	32.7	6.0	2.9	24.5	3.5	1.7	24.0	2.9	1.4
Qatar	22.6	4.0	1.9	17.3	2.8	1.4	10.0	1.9	1.0
Saudi Arabia	33.9	5.5	2.7	24.6	3.5	1.7	19.0	2.3	1.1
UAE	24.6	4.0	1.9	16.3	2.5	1.2	16.0	2.4	1.2
Total	32.0	4.9	—	22.7	3.0	—	18.1	2.2	—
West Asia									
Iraq	37.2	5.8	2.9	34.2	4.6	2.3	28.0	3.6	1.8
Jordan	34.3	5.0	2.4	28.5	3.6	1.8	27.0	3.4	1.6
Lebanon	22.6	2.7	1.3	16.7	2.0	1.0	15.0	1.8	0.9
Syria	35.5	5.3	2.6	29.5	4.0	1.9	24.0	2.9	1.4
Yemen	43.9	7.4	3.6	40.7	6.3	3.1	33.0	4.5	2.2
Total	37.4	5.5	—	33.4	4.3	—	27.7	3.3	—
Maghreb									
Algeria	30.2	4.3	2.1	19.6	2.3	1.1	17.0	1.7	0.9
Libya	29.4	4.6	2.3	22.3	2.9	1.4	23.0	2.9	1.4
Tunisia	24.9	3.2	1.5	16.8	2.0	1.0	17.0	2.0	1.0
Mauritania	41.7	5.7	2.8	37.0	5.0	2.5	33.0	4.2	2.1
Morocco	27.6	3.7	1.8	22.1	2.6	1.3	19.0	2.2	1.1
Total	28.8	3.6	—	21.0	2.3	—	18.8	2.0	—
Nile Valley African Horn									
Comoros	39.3	5.7	2.8	40.4	5.2	2.6	31.5	4.1	2.0
Djibouti	42.0	5.8	2.8	33.2	4.0	2.0	25.0	2.6	1.3
Egypt	27.9	3.7	1.8	26.9	3.4	1.6	24.0	2.9	1.4
Somalia	39.3	5.5	2.7	47.0	7.1	3.5	42.0	6.3	3.1
Sudan	44.6	6.3	3.1	40.1	5.6	2.7	36.0	4.8	2.3
Total	29.1	3.9	—	29.1	3.7	—	29.3	3.6	—
Grand Total	31.5	4.2	—	27.0	3.3	—	25.0	3.0	—

* Oman, 1993; Yemen, 1994; Egypt, 1996

### Mortality

The mortality indicators analyzed and explained here include the crude death rate, infant mortality rate, under-5 mortality rate, and expectation of life at birth. The crude death rate indicates the number of deaths per 1000 people for a particular period (say, a year); this remained high during 1992, especially in Somalia (59.5), Mauritania (14.8), Sudan (14.3), Comoros (11.9), Yemen (11.8), and Djibouti (11.4). Lower death rates were reported in GCC countries, along with Jordan and Syria in West Asia, the Maghreb countries except Mauritania, and Egypt in the Nile Valley African Horn (Table 5). A marked decline of 41.5 points in the crude death rate was noted in Somalia during 1992–2002, whereas a moderate declining trend was observed in all the other countries and sectors. The trend continued until 2012, except for some difference in Jordan, Algeria, Tunisia, and Morocco.

**Table 5:** Mortality Indicators.

Sectors and States	CDR			IMR			Under 5 MR			Life expectancy		
	1992*	2002	2012	1992*	2002	2012	1992*	2002	2012	1992*	2002	2012
GCC												
Bahrain	3.6	3	3	23.1	15.1	10	27.8	17.9	12	72.1	75.4	78.3
Kuwait	2.4	2.1	2	11.7	10.5	8	14.7	12.8	9	73.5	75.2	77.3
Oman	4.4	3.7	3	24.2	20.1	15	33.5	27.6	20	70	72	74.5
Qatar	2.3	2	2	13.4	8.7	7	19.1	12.1	9	73.7	76.2	78.1
Saudi Arabia	4.4	3.6	3	26.3	21.6	16	31.5	25.3	18	70.7	72.1	74.4
UAE	2.9	2.3	2	22.5	15.9	12	26.9	18.8	14	72	74.6	76.7
Total	4.1	3.2	2.7	—	—	—	—	—	—	—	—	—
West Asia												
Iraq	7.7	6	5	79.1	58.2	40	100.3	72.7	49	63.9	67.4	70.9
Jordan	3.2	2.4	3	33.7	20	16	41.1	22.5	18	76.3	79.3	80.2
Lebanon	6.1	6.1	7	32.1	21.9	15	35	23.9	17	69.9	72.8	75.2
Syria	4.8	4	4	30.2	21.2	15	36.8	25.5	18	69.5	72.4	74.9
Yemen	11.8	9.3	7	81.7	70.9	54	115.2	96.1	71	57.4	60.3	64.1
Total	7.7	6.1	5.3	—	—	—	—	—	—	—	—	—
Maghreb												
Algeria	5.2	4.4	5	41.6	35.8	25	47.4	40.4	29	70.7	73	76
Libya	5.1	3.8	3	32.4	18	19	39.1	20.4	22	69.2	73.3	75.6
Tunisia	5.5	5.5	6	89.8	74.5	59	155.8	117.4	89	50.3	57.6	61.5
Mauritania	14.8	10.5	9	59.7	39	26	74.4	45.8	31	68.9	77	76.1
Morocco	6.1	4.8	5	44.8	35.4	25	58.2	45.2	31	71.2	73	75.2
Total	5.9	4.9	5.1	—	—	—	—	—	—	—	—	—
Nile Valley African Horn												
Comoros	11.9	10.4	8.2	101.7	86.9	69	144.6	86.9	94.5	56.6	59.3	62.7
Djibouti	11.4	9.5	8	89.1	70.4	53	123.1	96.7	72	56.2	58.5	61.6
Egypt	5.8	5.1	5	49	35.9	24	61.5	44.2	29	67.4	70.1	72.9
Somalia	59.5	18	15	187.1	122	104	309.8	205.5	170	17.2	47	50.8
Sudan	14.3	12.9	10	91.7	86.3	64	154.1	143.5	106	51.1	52.4	62.6
Total	10.8	6.5	7.4	—	—	—	—	—	—	—	—	—
Grand Total	7.7	5.5	5.8	—	—	—	—	—	—	—	—	—

* Oman, 1993; Yemen, 1994; Egypt, 1996

**Fig 2:**
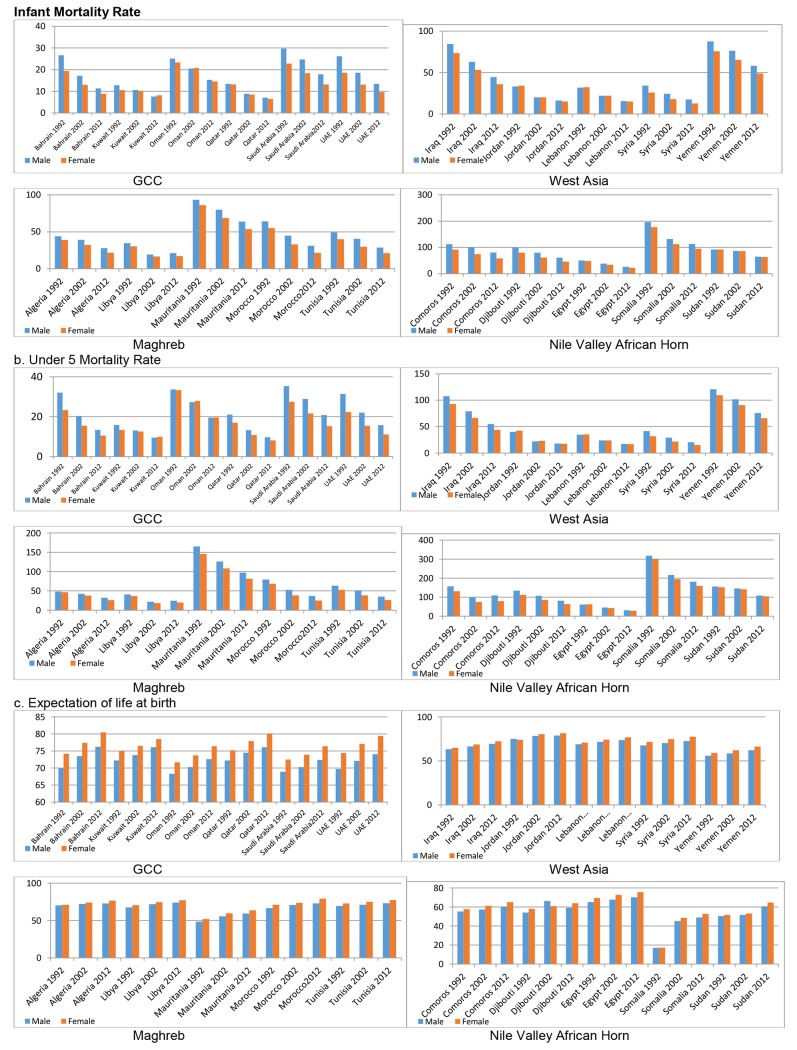
Male Female differences in mortality by sectors and states.

The IMR has remained low in all GCC countries since 1992, with the rate kept below 30 for both males and females; it was brought further down to below 25 in 2002 and thereafter to below 20 in 2012 (Fig 2). West Asian countries, such as Iraq and Yemen, registered higher IMRs in 1992, but these went down in 2002 and 2012. Jordan, Lebanon, and Syria have had low IMRs since 1992, which declined further in 2002 and 2012. All the countries except Iraq showed a sharp decline in infant mortality, which was directly related to population size, annual total births, low birth weight, and maternal mortality. The decline may also be attributed to an inverse relationship between infant mortality and literacy, gross national product, access to safe drinking water, and adequate sanitary facilities [8].

Mauritania, followed by Morocco, had the highest IMR in the Maghreb sector, but the rate is slowly declining. The Nile Valley African Horn countries, namely, Somalia, Comoros, Djibouti, and Sudan, showed significantly higher IMRs but with a declining trend. An unimpressive IMR decline was reported in Somalia, Comoros, and Sudan.

The mortality rate of children under 5 years of age in the Arab world is the subject of debate because it stands higher (except GCC) than that in developed countries [38]. Maternal and child health care components influence under-5 mortality, with higher parities, combined with less care during infancy, leading to higher incidences. The rate remained high in the West Asian nations of Iraq and Yemen, the Maghreb country of Mauritania, and the Nile Valley African Horn countries of Comoros, Djibouti, Somalia, and Sudan during 1992. An unimpressive decline in under-5 mortality was noted during 1992–2002, whereas a significant reduction in line with the Millennium Development Goals was recorded during 2002–2012, except in Somalia and Sudan [34,35].

One indicator of health improvement, namely, expectation of life at birth, stands high in the Arab world, possibly due to cleanliness, dietary habits, activity profile, and lifestyle. All countries in the GCC have had a life expectancy above 70 years for both males and females since 1992. Life expectancy increased over time by at least 2 years in each decade, both for males and females. West Asian countries also maintained health conditions at par with GCC standards, which kept their life expectancy levels comparable during 2012, except in Yemen, which lagged behind in this variable, showing a poor situation during 1992, coupled with a slow pace of progress. The Maghreb countries, except Mauritania, had high life expectancy rates during 2002 and 2012. The Nile Valley African Horn countries had low levels of life expectancy in all three time periods; Egypt was the only country in this sector with a high life expectancy since 1992.

The Arab world, which is characterized by frequent migrations—within the state, outside the state but from another Arab state, and outside the Arab states, have varying influences, particularly socioeconomic [39,40] and health [41,42]. Most infectious diseases in the region, including sexually transmitted diseases and precipitating conditions, are results of migration and migrant status.

## Conclusions and Recommendations

The population of the Arab world has grown remarkably. The growth accelerated in the millennium due to higher natural increase and migration. Whereas the wide gap between fertility and mortality levels led to an increase in national populations, the accelerated development activities in relation to the recovery of petroleum reserves attracted immigrant populations; together, these caused the population to grow at a faster rate. The vibrant Arab world, with its accelerated development, adoption of technological innovations, and huge investments in housing, education, health, and public services infrastructure, thus paved the way for an efficient transition on the demographic front, at a speed that no other region has witnessed. The demographic transition in the Arab world followed a path similar to that in other developing countries, although it took a long time for fertility reduction to take place. The trend of fertility decline has reduced the growth rate rapidly, as seen in countries such as Lebanon. Countries with higher reductions in fertility are expected to achieve below-replacement level soon, with other nations set to follow the trend in the future. In addition, changing labor laws and immigration policies in the GCC regulate the flow of migrants from Asian and African countries, further bridging the youth bulge and age-sex structure. Thus, the Arab world is expected to gain demographic stability soon, which is conducive for further progress and gains.

The comparatively higher number of births than deaths results in geometric additions to the population, as evident in a large part of the Arab world, where there is high natural growth due to low levels of mortality. The natural growth rate has declined gradually along with the decrease in fertility, peaking at the turn of the millennium and then accelerating gradually. The demographic transition in the Arab world is also expected to impact East Asian developing countries soon, with changing labor policies and immigration laws in host countries in the Arab world further restricting new entries, thereby reducing net migration. These factors together contribute to population stabilization in the Arab world, enabling the region to witness a new demographic trend in the near future.

The rapidly improving living conditions in the Arab world add years to life through their impacts on vital indicators of fertility and mortality. The population increase through natural growth and net migration also enhances living conditions. Thus, the bigger the population, the better the quality of life, as shown by urbanization trends in various parts of the region. Urban centers are better equipped in terms of housing, water, electricity, and sewage services, as well as educational, employment, health, and public utility infrastructure. All these improvements enable the Arab world to embark on a new era of demographic transition characterized by an accelerated decline in fertility and reductions in mortality due to poverty, malnutrition, infectious diseases, and public health casualties.

## Supporting Information

S1 File Data sets downloaded from www.Census.gov and calculations done for this research showing Population Size for 1992, 2002 and 2012 (Table 1); Growth rates calculations (Table 2); Components of poulation growth calculations (Table 3). For details of Table 4 visit www.census.gov/hhes/fertility and for details of Table 5 visit www.census.gov/health.
(XLSX)
